# Revealing layer-specific cortical activity in human M1 using high-resolution line-scanning fMRI

**DOI:** 10.1162/imag_a_00477

**Published:** 2025-02-21

**Authors:** Nils Nothnagel, A. Tyler Morgan, Lars Muckli, Jozien Goense

**Affiliations:** School of Psychology & Neuroscience, University of Glasgow, Glasgow, United Kingdom; Functional MRI Facility, National Institute of Mental Health, Bethesda, MD, United States; Beckman Institute for Advanced Science and Technology, University of Illinois, Urbana-Champaign, Urbana, IL, United States; Department of Psychology, University of Illinois, Urbana-Champaign, Champaign, IL, United States; Department of Bioengineering, University of Illinois, Urbana-Champaign, Urbana, IL, United States; Neuroscience Program, University of Illinois, Urbana-Champaign, Urbana, IL, United States

**Keywords:** line scanning, high-resolution fMRI, cortical layers, hemodynamic response, negative BOLD

## Abstract

In recent years, ultra-high field functional MRI has allowed researchers to study cortical activity at high spatiotemporal resolution. Advancements in technology have made it possible to perform fMRI of cortical laminae, which is crucial for understanding and mapping of local circuits and overall brain function. Unlike invasive electrophysiology, fMRI provides a non-invasive approach to studying human and animal brain function. However, achieving high spatial resolution has often meant sacrificing temporal resolution. In contrast, line-scanning fMRI maintains both high spatial and temporal resolution, and has been successfully applied to animals to detect laminar differences of the hemodynamic response. Although this method has been extended to human brain imaging in initial studies, staying within SAR safety limits while maintaining a well-defined saturation profile at a short TR is a major challenge. We present a method for gradient-echo-based human line-scanning that uses four saturation regions to achieve a line with narrow FWHM (3.9 mm) at high spatiotemporal resolution (voxel size 0.39 x 3.0 x 3.0 mm^3^, TR = 250 ms). We demonstrate its use for laminar fMRI by measuring laminar time courses in the hand knob of the primary human motor cortex during a finger-tapping task. Our findings indicate differences in the onset and temporal characteristics of the hemodynamic response across cortical layers. Deeper layers exhibited distinct temporal dynamics compared with the gray matter near the cortical surface. Specifically, the BOLD response reached 95% of the maximum amplitude earlier than the superficial layers, and demonstrated a faster return to baseline after stimulus offset. We demonstrate that line-scanning fMRI offers a valuable tool for investigating recordings at a very high temporal and spatial resolution and could help advance our understanding of the mechanistic nature of the BOLD response.

## 1 Introduction

Ultra-high-field functional MRI (fMRI) in humans has enabled the study of cortical activity at very high spatiotemporal resolutions (Guidi et al., 2016;Huber et al., 2015;Jia et al., 2023;Shao et al., 2021;Shen et al., 2008;Siero et al., 2013). These advances have enabled researchers to perform functional MRI of cortical laminae, which can disentangle local circuits (Finn et al., 2019;Goense, 2018;Huber, Finn, et al., 2021) with implications for studying brain-wide function. Unlike electrophysiology, which is considered the gold standard for measuring neural activity (Self et al., 2019), fMRI is completely non-invasive, making it broadly accessible for studying brain function in humans and animals. As it is based on a hemodynamic response, fMRI is an indirect measure of cortical activity.

The most commonly used sequence in fMRI is the gradient-echo-BOLD (GE-BOLD) sequence due to its ease of application, its strong signal changes, and its sampling efficiency. However, GE-BOLD is sensitive to various physiological parameters such as local changes in cerebral blood flow (CBF), cerebral blood volume (CBV), and oxygen consumption (CMRO2). GE-BOLD shows a bias toward the draining vasculature, which limits its precision in mapping the exact location of cortical activity. Non-BOLD sequences have gained traction because they measure specific aspects of neural activity such as CBF (Kashyap et al., 2021) or CMRO2 (Shao et al., 2021), but due to their complex and often custom implementation, the most commonly used contrast is still the GE-BOLD contrast (Bergmann et al., 2024;Fracasso et al., 2018;Huber et al., 2019;Nothnagel et al., 2023)

The pursuit of high spatial resolution usually requires compromising temporal resolution and/or spatial coverage. To achieve high spatial resolution (voxel size <0.8 mm), the field-of-view (FOV)/imaging slab is reduced to contain the area of interest (10–20 cm), and repetition times (TR) are often long, with reported TRs of up to 9 s (Koiso et al., 2023), although the current standard is 2–3 s.

Simultaneously achieving high spatial and temporal resolution can be achieved via a compromise of reducing coverage. By minimizing both spatial (voxel size <0.8 mm) and temporal (TR <1 s) resolution, dimensions tangential to the cortical surface can be collapsed into a single line.Yu et al. (2014)implemented a line-scanning fMRI method with very high temporal (TR = 50 ms) and spatial (voxel size 50 μm) resolutions and used a sensory stimulation paradigm to elicit functional responses in rat sensory and motor cortex. They detected laminar differences in the BOLD time courses, with the hemodynamic response being initiated in layer IV of sensory cortex and layers II/III and V of motor cortex in rats. Further improvements and cross-validations followed the original investigation.Albers et al. (2018)compared sensory and optogenetically induced BOLD responses using line-scanning fMRI in rats and confirmed that the method can detect time differences in the onsets in the range of 100 ms.Nunes et al. (2019,^2021^) introduced diffusion-weighted line-scanning fMRI and demonstrated its ability to detect early onsets (<100 ms) of the functional signal in the middle layers of rat sensory cortex.Choi, Zeng, et al. (2022)andChoi et al. (2023)presented a method for bilateral line scanning and detected laminar patterns of resting-state signals. Recent work has also demonstrated the possibility of functional line scanning using GRASE (Choi, Yu, et al., 2022) and spin-echo-BOLD readouts (Choi et al., 2024).

Line-scanning fMRI has recently been applied to humans (Balasubramanian et al., 2021;Morgan et al., 2020;Raimondo et al., 2021, 2022;Raimondo, Priovoulos, et al., 2023). One of the major challenges of applying line scanning to humans is staying within SAR safety limits while maintaining a well-defined saturation profile at a short TR.Raimondo et al. (2021)reported their initial results and measured GE-BOLD signals with high spatial (200 μm) and temporal (TR = 200 ms) resolution using two saturation bands. However, this approach produced a line profile with a full-width-half-maximum (FWHM) of ~6 mm, which limits the accuracy of laminar BOLD studies when the spatial extent of the activation is smaller than this distance, as signals from adjacent areas would be projected into the line.Heij et al. (2023)highlighted the importance of planning the line location in advance, as the highly convoluted human cortex makes manual line placement less accurate, particularly when aiming for the line to pass exactly radially through the cortical surface.Balasubramanian et al. (2021)presented a line-scanning method for acquiring layer-specific diffusion imaging and demonstrated that they can measure diffusion differences in the cortical layers of M1 and S1 with a 250–500 μm radial resolution.

Here, we demonstrate GE line scanning for layer-specific human brain imaging with a very sharp and thin line profile (3 mm). This makes the method suitable for application to smaller (tangential) activations, such as digit maps in S1 or the hand knob in M1. Line scanning is well suited for cortical depth-specific functional imaging due to its very high resolution in the laminar dimension while maintaining high signal-to-noise ratio (SNR). At a laminar resolution of 0.39 mm, the line scan voxel of 0.39 x 3.0 x 3.0 mm^3^captures the same number of protons as an equivalent isotropic voxel of (1.5 mm)^3^. The line scan voxel integrates signals from the same cortical depth, therefore, reducing partial volume effects and increasing temporal SNR (tSNR) (Blazejewska et al., 2019;Kiebel et al., 2000).

Our GE-line-scanning method involves four saturation regions to create a sharp line profile at acceptable SAR levels. We apply the method to obtain laminar positive and negative BOLD time courses in the primary motor cortex during a finger-tapping task. This stimulus was chosen as it produces a broad activity in the hand knob in M1 and is a common task for human laminar fMRI studies in M1 (Chai et al., 2020;Guidi et al., 2016;Huber et al., 2017;Persichetti et al., 2020;Shao et al., 2022). We find that the time courses of both positive and negative BOLD show laminar specificity. Additionally, we describe a method for accurately segmenting the gray matter from line-scanning acquisitions using both the magnitude and phase information from accompanying high-resolution FLASH scans. We also detail the process of preparing and executing our GE line-scanning sequence and the post-processing, with the overall goal of further advancing the human line-scanning fMRI method.

## 2 Methods

**Fig. 1.**
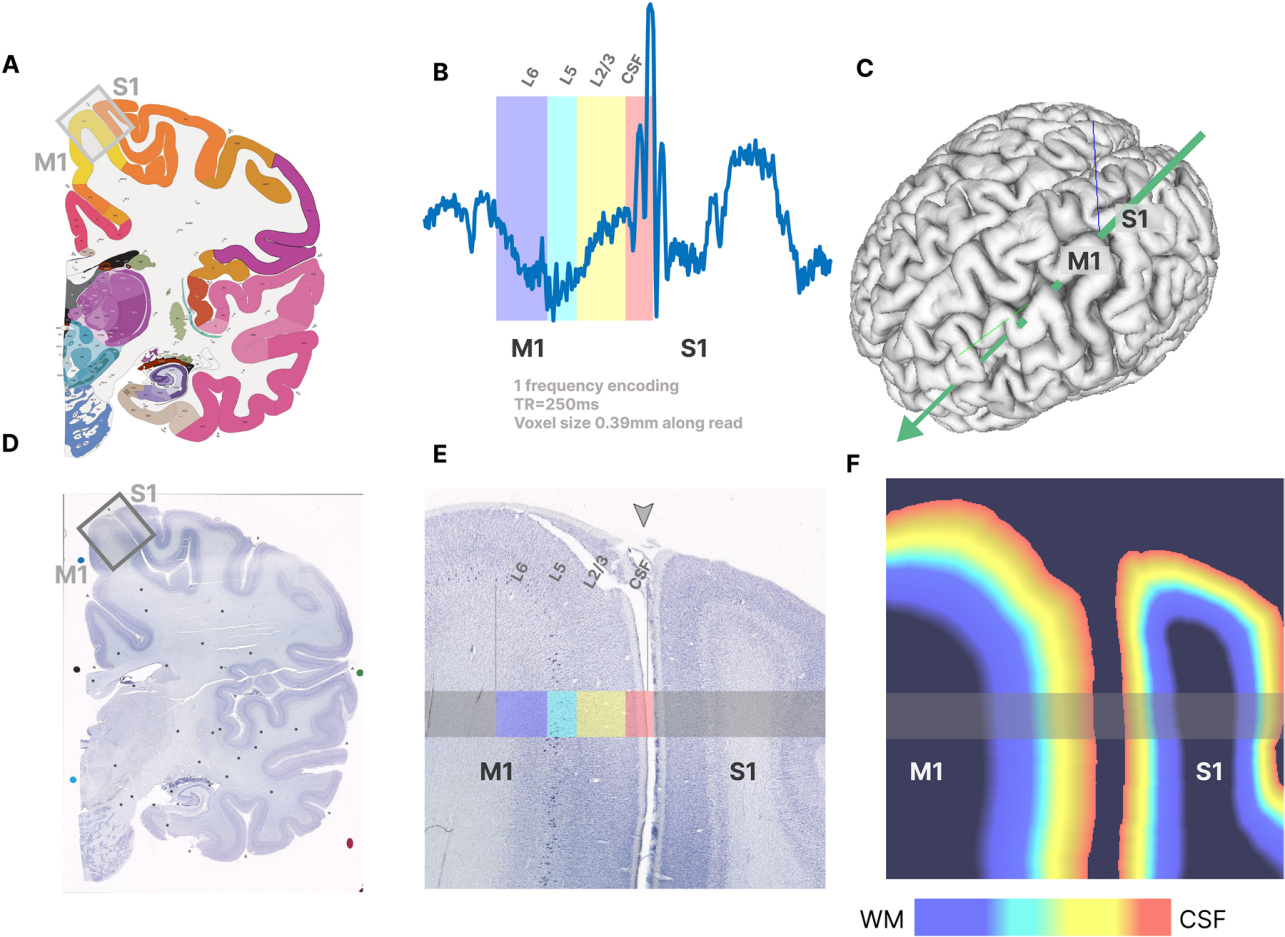
Panels (A, C, D) show the location of sensorimotor cortex and panel (B) displays the 1D profile of a line that passes through the layers of the human primary motor cortex (M1) and the primary sensory cortex (S1). Panel (C) shows the location and orientation of a line passing through the hand knob of M1. Panels (E and F) display the underlying microscopy image of the M1/S1 region. The definition of cortical layers in M1 is marked in colors and is based onPalomero-Gallagher and Zilles (2019), showing a thick layer 6, a thinner layer 5, an absence of layer 4, and a thick layer 2/3. Panel (E) shows how the line from Panel B passes radially through the layers of M1. The underlying microscopy image and the atlas are taken from the Allen 34 years old Human Brain Atlas (Allen Institute for Brain Science,https://atlas.brain-map.org/andhttps://atlas.brain-map.org/atlas?atlas=265297126,Ding et al., 2016;Hawrylycz et al., 2012). Panel (F) indicates the cortical depth estimation for the image of Panel (E).

We implemented a multi-echo GE-based line-scanning sequence and demonstrated its use in non-invasively recording layer-specific functional activity in primary motor cortex (M1). We collapse the entire MRI acquisition into a single one-dimensional readout line through the postcentral gyrus (Fig. 1C). We use a dedicated alignment procedure (seesections 2.3and2.4) to ensure that this line passes through the cortex of M1 at a specific, targeted location (Fig. 1C-F).Figure 1Billustrates the amplitude of the collapsed signal andFigure 1C-Fillustrates the laminar arrangement of M1 and S1, providing a visual representation of this process.

### 2.1 Implementation of the line-scanning sequence

The line-scanning sequence was based on a multi-echo FLASH (fast low-angle shot) pulse sequence on a 7T Siemens Magnetom Terra (Siemens Healthineers, Germany), incorporating saturation pulses to select the line (seeFig. 2). The signal saturation was achieved with four custom Shinnar-Le Roux (SLR) saturation pulses. Two inner SLR pulses were created using the*sigpy*package (Ong & Lustig, 2019) with N = 256 samples; passband ripple 0.01%; stopband ripple 0.1%; maximum phase; flip angle 110˚; bandwidth-time product 19; 7800 µs pulse duration; thickness 15 mm. Two outer SLR pulses were created also using*sigpy*with N = 256 samples; passband ripple 0.01%; stopband ripple 0.1%; maximum phase; flip angle 120˚; bandwidth-time product 2.7; 3800 µs pulse duration; thickness 120 mm. We applied root flipping to lower the B1 peak power of all SLR pulses. We positioned the inner SLR pulse symmetrically around the center to create a well-defined line profile in the center of the slice. We positioned the outer SLR pulse on the rest of the brain tissue to suppress the remaining signal. The minimum TR was 109 ms but we used 250 ms to reduce SAR and increase signal-to-noise (SNR). We expected this TR to capture the essential properties of the dynamics of the hemodynamic response.

**Fig. 2.**
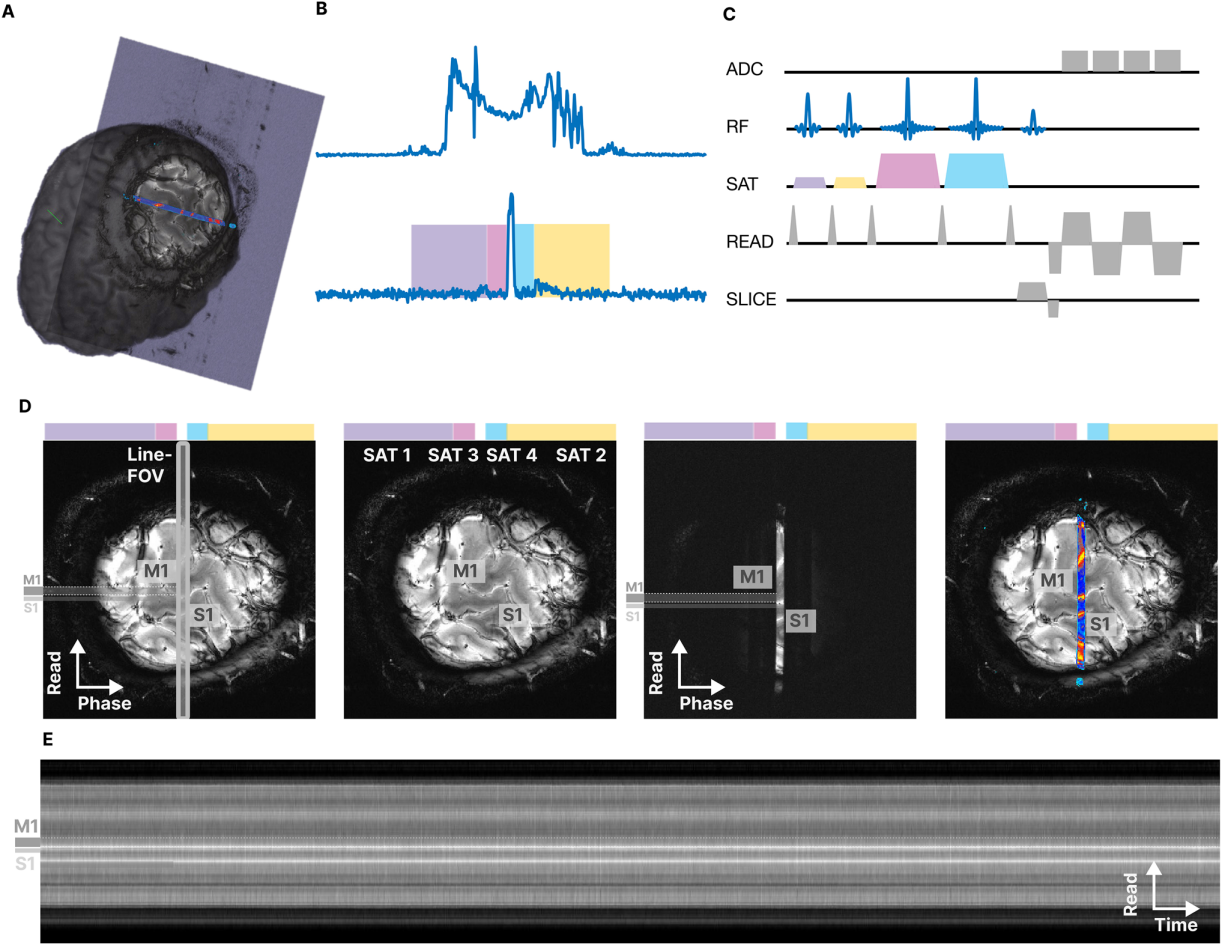
The line-scanning sequence. Panel (A) shows the location of a double-oblique slice containing the line. It includes the hand knob area of M1, S1, with the center line of the slice passing perpendicularly through M1 at the target location. Panel (B) illustrates the arrangement of the four saturation pulses (colors) to suppress all signals in the slice except for the center line. Two outer saturation pulses (purple and yellow) suppress the signal from the bulk, while two narrow saturation regions with a sharp edge (blue and pink) create a sharp gap of 3 mm in the center of the slice for the line. Panel (C) shows the pulse diagram with the timing of the RF pulses and gradients, and the readout gradients for an acquisition with four echoes. Panel (D) consists of four images: the first image shows the acquired slice with the line indicated in the center (x-axis: phase encoding direction, y-axis: readout direction). The second image indicates the location of M1 and S1, and the position of the four saturation regions (colored regions). The third image displays the image with the saturation pulses turned on, demonstrating that all the signal is suppressed except for the targeted center line of the slice. The last image overlays the third image (saturation on) onto the first image (saturation off). Panel (E) shows the functional time courses of a 4-min line scan of all voxels in the center line (x-axis: time domain, y-axis: readout direction).

We compared this implementation on a regular FLASH image with a conventional saturation strategy by placing vendor-implemented saturation bands to create the line, with otherwise matching parameters. For all approaches, we measured SAR levels as taken from the log files of the scanner. We used MATLAB to calculate the FWHM of each approach taking the average of 10 center profiles on the FLASH images recorded with saturation pulses (seeFig. 5B). We calculated a leakage as percentage as the fraction of signal generated from voxels outside the line, divided by the entire signal of all voxels.

In the functional experiment, our proposed sequence allows for the option to turn the phase-encoding gradient off. The sequence offers several choices:

1. A regular FLASH (Fig. 2D) to provide an anatomical reference that is used for segmenting the gray matter in the area of interest (seesection 2.6.5).
2. A FLASH with saturation bands. This shows the target line of 3 mm in the center of the slice (Fig. 2D, third panel), and was used to assess signal suppression quality and to confirm the absence of artifacts that would be projected into the line.
3. Line scanning with saturation pulses on and phase-encoding switched off (Fig. 2E). This turns the sequence into a one-dimensional recording at the center of the slice. We used this option for the functional runs.

The sequence parameters of these scans can be found inTable 1.

**Table 1.** Parameters of the different sequences.

	3T Anatomy	7T Anatomy	Online GLM	Confirm location	2D Anat. reference	Functional line scan
Sequence	MPRAGE	MP2RAGE	EPI	FLASH	FLASH	FLASH
Voxel size(mm ^3^ )	1.0 x 1.0 x 1.0	0.63 x 0.63 x 0.63	1.0 x 1.0 x 3.0	1.2 x 1.2 x 3.0	0.39 x 0.39 x 3.0	0.39 x 3.0 x 3.0
FOV (mm ^3^ ) read-phase-slice	256 x 256 x 192	240 x 225 x 160	150 x 150 x 3	224 x 224 x 3	224 x 224 x 3	224 x 3 x 3
TR (ms)	2600	4680	2000	250	250	250
TE (ms)	40	2.09	25	12	14,25,36,47	14,25,36,47
FA	8°	5°/6°	68°	15°	15°	15°
IPat	2	3	3	1	1	1
Has phase encoding?	Yes	Yes	Yes	yes	yes	no

### 2.2 Volunteer recruitment

We recruited 14 healthy volunteers for this study. We excluded two participants because we could not find a sufficiently flat stretch in their motor cortex to position the line (seesection 2.3) and one participant because of head motion. We conducted functional line scanning on 12 healthy volunteers (7/5 m/f, age range 21–35 years, 10 right handed, 1 left handed, 1 ambidextrous). Before their participation, we obtained informed consent from each volunteer. The study was reviewed and approved by the local ethics committee of the College of Medical, Veterinary and Life Sciences at the University of Glasgow (Project no. 302122). We applied standard site-specific inclusion criteria, including the absence of metal in the body, age over 18 years, normal or corrected-to-normal vision, normal hearing, and no diagnosis of neurological or mental health disorders. These criteria were confirmed during the participants’ initial visit to the facility.

### 2.3 Pre-session slice planning

**Fig. 3.**
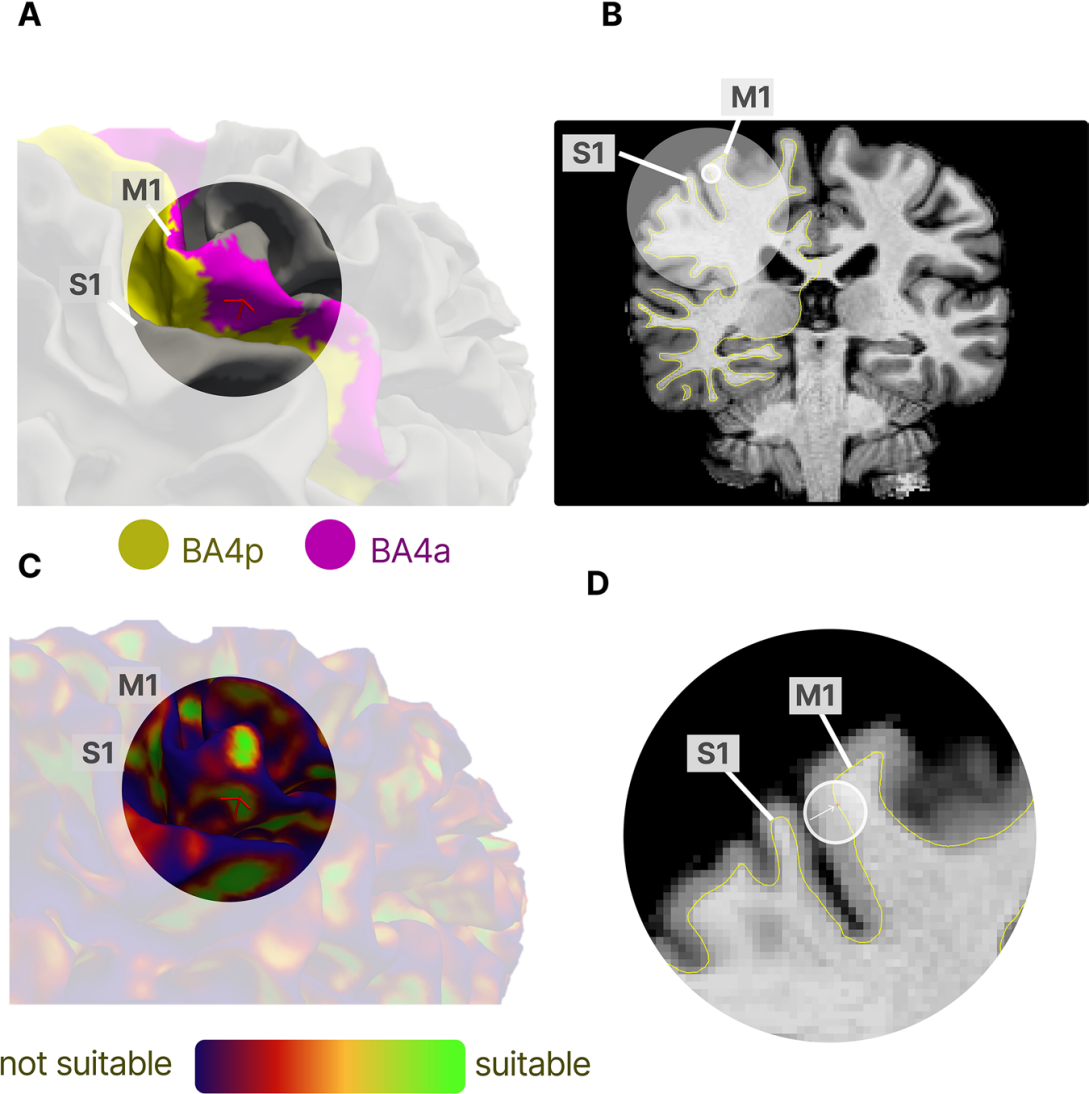
Illustration of the procedure for identification of a suitable location for line scanning in primary motor cortex. Panel (A) indicates the hand area in the anterior primary motor cortex (Brodmann Area 4a, BA4a, pink) on the cortical white matter surface. Panel (B) shows the location of M1/S1 and the target location on the MPRAGE anatomical scan. Panel (C) shows the same cortical surface as in Panel A but with the curvature value for each vertex. We chose as suitable location an area with low curvature (green) within the hand area of BA4a. Panel (D) zooms in on the structural scan of Panel (B) and marks the selected vertex in M1 in voxel space.

Before the 7T session, we identified a flat area in the hand region of the primary motor cortex (M1) on either the left or the right hemisphere (seeFig. 3Aand3B) at 3T using data acquired earlier at 3T (Tim Trio MRI System, Siemens Healthineers, Erlangen, Germany). We acquired an anatomical scan (MPRAGE, seeTable 1) to create a cortical reconstruction using Freesurfer 7.3 (*recon-all*) (Fischl, 2012).Figure 3shows how we use Freesurfer’s probability atlas to narrow down our search for the anterior primary motor cortex to Brodmann Area 4a (BA4a) on the white matter surface (Huber et al., 2017). We then refined the search within inside BA4a for a sufficiently flat spot following the method described inMorgan et al. (2020).Figure 3Cindicates the curvature of the cortex with highlighted area over BA4a. Green areas indicate low curvature (suitable for line scanning) and red and blue areas indicate high curvature (unsuitable for line scanning).

After identifying a suitable location in BA4a, we usedMorgan et al.’s (2020)method to calculate coordinates for a slice that contains the line in its center. Given that we could rotate the slice containing the line by 360º around the cortical surface normal, we selected an angle that led to a slice with the least amount of signal outside the line in the image. This procedure led consistently to a double-oblique coronal slice, tilted 20º–40º toward the axial plane and approximately 2º–10º away from the sagittal plane. If no flat area could be found, we did not proceed with the volunteer (we were not able to find a flat spot in two participants based on this criterion).

### 2.4 Setup at the scanner

For the functional line-scanning experiments, we used a 7T Magnetom Terra scanner (Siemens Healthineers, Erlangen, Germany) equipped with a 32-channel head coil (Nova Technologies). After obtaining an anatomical scan (MP2RAGE, seeTable 1) at the beginning of the session, we exported the DICOM images to an external computer. We then converted the images to NifTI format using*dicom2niix*(Li et al., 2016) and applied LAYNIIs*LN_MPRAGE_DNOISE*tool to denoise the MP2RAGE background (Huber, Poser, et al., 2021). Next, we computed a rigid 6-degree-of-freedom transformation in ITK-SNAP (Yushkevich et al., 2006) to find a transformation between the head position during the pre-session anatomical scan and the current head position in the scanner (seeTable 1). We then applied the alignment procedure ofMorgan et al. (2020)to obtain the coordinates of the new slice for the current head position (seesection 2.3). We then typed in the new slice coordinates as three positions for the location, two angles for the double-oblique slice orientation, and the in-plane slice angle. We confirmed the new slice location using the procedure outlined inFigure 4.

**Fig. 4.**
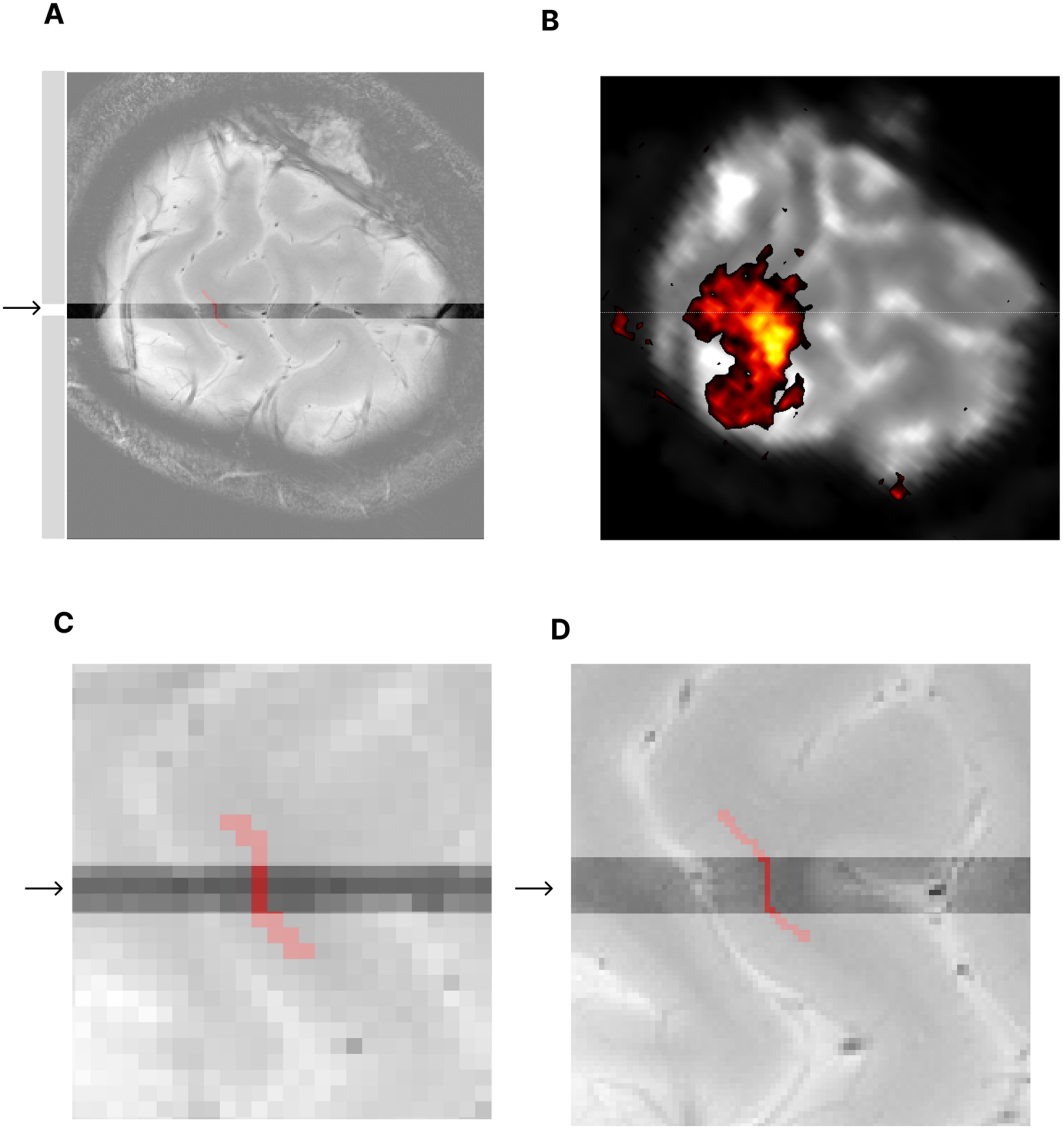
Illustration of the procedure for planning the target line for scanning. Panel (A) shows a high-resolution FLASH scan with the line passing through the center from left to right. The outer regions are shaded out since they will be saturated in the line scan. The top row in Panel (B) shows the result of an online GLM of a low-resolution EPI (1.0 x 1.0 x 3.0 mm^3^) to confirm BOLD activation in the patch. Panels (C and D) zoom in on the target location of a low-resolution (1.2 x 1.2 x 3.0 mm^3^) (C) and high-resolution FLASH (0.39 x 0.39 x 3.0 mm^3^) (D). The red line shows the WM surface segmentation (red) around the target spot. This is to visualize how the line passes perpendicularly through the cortex, as indicated by the red line showing the GM/WM boundary. While it appears on the low-resolution FLASH as if the line does not pass perpendicular WM at the identified line-scan position, the smaller voxels and better contrast of the high-resolution FLASH reveal a small, 3–4 mm straight patch between a convex and a concave region that we targeted in our selection process (seesection 2.3).

To confirm that the line passes through M1 radially, we acquired a low-resolution 2D FLASH at the new slice location (Fig. 4C). This acquisition had an additional slice excitation pulse that was rotated by 90º around the center line to cast a shadow at the location of the target line (seeTable 1). This created a visual guide to confirm that the center line passes radially through M1, and allowed for small adjustments if needed. If the slice did not seem aligned, we would repeat the MP2RAGE and repeat the process. If it still did not work, we would end the session. If we were at the right location, we would then run a single-slice functional EPI scan at the target slice (seeTable 1) with a finger-tapping paradigm of the contralateral hand (described insection 2.6) and use an online GLM to confirm functional activity at the target location (Fig. 4B).

We performed an automated B0-based shimming procedure for a region that contains the slice and a minimum thickness of 90 mm (Nassirpour et al., 2018). Next, we would acquire a B1 map and adjust the transmit voltage using a region of interest (ROI) in the motor area. For anatomical reference, we would then acquire a high-resolution 2D FLASH scan at this location (seesection 2.3; parameters inTable 1), with and without saturation pulses (Fig. 2D). We then started the functional experiment of four functional line scans (two contralateral tapping and two ipsilateral tapping, with contralateral first).

### 2.5 Finger-tapping paradigm

We used a finger-tapping task using a block design, which elicits a strong BOLD response in M1 and is a standard paradigm for motor tasks (Chai et al., 2020;Guidi et al., 2016;Huber et al., 2017,^2019^;Persichetti et al., 2020;Shao et al., 2022). Each block consisted of 20 s of tapping followed by 20 s of rest, starting with a baseline. There were eight trials in total for each run. The participants could see instructions on the screen. During the baseline period, a cross was displayed, and participants were told to relax. During the tapping period, a message instructed them to tap with either their left or right hand. Participants were asked to tap only with their left hand or only with their right hand for the entire run. If the participant could remain still, we recorded at least four runs: two with the contralateral hand, then two with the ipsilateral hand. If there was time left at the end of the session, we would acquire an additional run for contralateral tapping (this was the case for three participants). Before the functional MRI scan, we practised the tapping motion outside of the scanner with the participants. We instructed the participants to tap four fingers simultaneously against the thumb, at a self-chosen pace.

### 2.6 Data processing

#### 2.6.1 Exporting and reading raw data

The raw data for all custom line-scanning acquisitions were exported from the scanner using the vendor-specific tool (*twix*). Subsequently, the data were transferred to an external computer and then converted from the vendor-specific .dat format into regular data arrays using the*pymapVBVD*tool, a python port of Philips Ehses’ MATLAB tool*mapVBVD*(https://github.com/wtclarke/pymapvbvd).

#### 2.6.2 Offline data reconstruction

The structural 2D data were transformed to the image domain using a 2D FFT along the read and phase dimensions. Then, a coil sensitivity map was calculated for the slice where the line was located using the 2D FLASH scan without signal saturation, employing BART’s*ecalib*tool (Uecker et al., 2015). Subsequently, the complex coil images of both structural scans (saturated and non-saturated) were combined using a SENSE-1 coil combination. The phase data were unwarped using the*skikit*package. Both the magnitude and phase data were stored for further analysis to create an anatomical reference (seesection 2.6.5).

The 1D functional data were transformed to the image domain using a 1D FFT along the readout dimension. Then, a root-of-sum-of-squares (RSS) coil combination was applied to reduce the array along the channel dimension. The data were then combined along the echo dimension using an optimal weighting suggested byKundu et al. (2012)with a target T_2_^*^of 25 ms using the formula

$$\text{weights}={\text{TE}}^{\text{*}}\text{exp}\left(-\frac{\text{TE}}{{\text{T}}_{2}^{\;\text{*}}}\right).$$

The final data formed a two-dimensional array, with the first dimension covering all voxels along the line and the second dimension covering all TRs in the functional run (Fig. 2E).

#### 2.6.3 Alignment of functional runs

We aligned all functional runs individually to the collapsed structural FLASH (with saturation) to ensure consistent activation location across all runs. We chose not to use sub-voxel shifts to avoid potential interpolation-induced blurring. Instead, we calculated the number of voxels to shift each run based on a simple numerical approach to find the highest cross-correlation to the collapsed scan. In our numerical search, we used 30 voxels surrounding the target gray matter in the M1–S1 region and then shifted each functional run by this number of voxels. This ensured that all the gray matter voxels in the functional runs aligned with the collapsed profile where the segmentation was defined (seesection 2.6.5).

We used the output of the alignment to detect scans that showed excessive head motion. Since a line scan only provides information about one of the six possible degrees of freedom for motion (the readout dimension), we could not apply retrospective motion correction. Any head motion after the alignment process meant that the signal was recorded from a cortical area different from the intended one. Additionally, the steady state was temporarily affected in the event of head motion which influenced the functional signal until it recovered. Therefore, we chose motion detection over motion correction. Our rule was: If we detected a shift during the alignment of the brightest voxel in the first echo inside the brain of more than three voxels over the entire run, we would exclude the entire run.

Across the 12 participants, we had to reject 12 out of 27 runs for contralateral (44%) and 16 out of 22 runs for ipsilateral (72%) tapping based on the above defined criterion. Most of the rejected scans were at the end of a session. The final subject pool consisted of eight participants with good data from both contralateral and ipsilateral tapping, three participants with good data from contralateral tapping only, and one participant with no acceptable data.

#### 2.6.4 Storing data in NifTI format

After the alignment, all functional data were stored in NifTI format using the*nibabel*package (Brett et al., 2023). The phase image of the profile scan was also stored separately for segmentation later on (seesection 2.6.5). The slice orientation information was obtained for all scans from the original DICOM after conversion to NifTI using the*dicom2niix*package (https://github.com/rordenlab/dcm2niix).

#### 2.6.5 Location of the postcentral sulcus in the line and GM segmentation

**Fig. 5.**
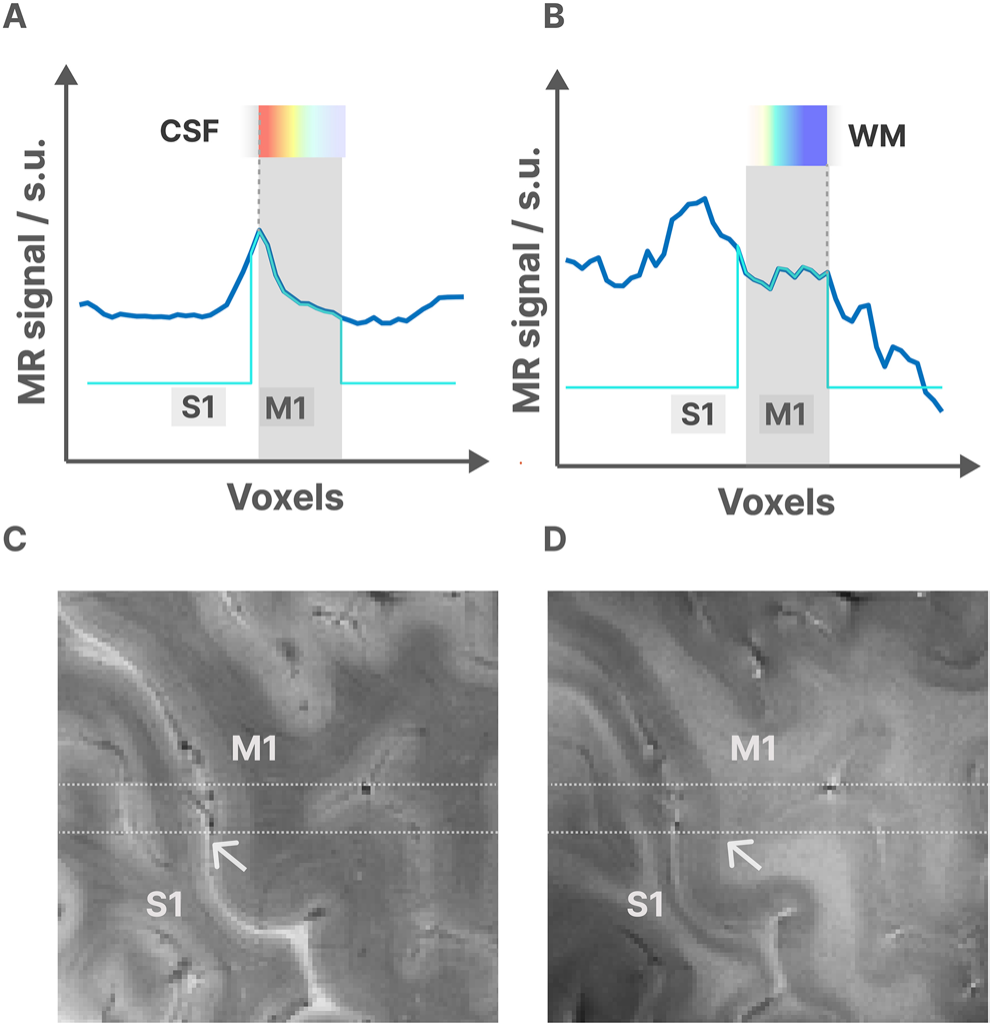
shows the definition of the gray matter boundaries in the magnitude (A, C) and phase (B, D) images. Panel (A) shows the magnitude profile of the line passing through M1/S1, with the gray matter voxels marked. The brightest voxel in this profile indicates the CSF border. Panel (C) shows the magnitude of the high-resolution 2D FLASH image. The line is marked in the center. Panel (B) shows the phase of the line, with the gray matter voxels marked. The white matter border is indicated by the location where the phase slightly jumps at the gray/white matter border due to a T2* change, and we used this jump to define the white matter border. Panel (D) shows the high-resolution 2D FLASH phase image.

First, we started by drawing an initial segmentation of the gray matter on the magnitude image of the profile scan using ITK-SNAP and saved the result in NIfTI format. Next, we collapsed the center line of this segmentation into a 1D line, which should closely label the target gray matter in the line scan data. However, in practice, it may not be perfect. To find a consistent upper border between gray matter and cerebrospinal fluid (CSF), we applied the following method illustrated inFigure 5. We searched for the brightest voxels near one of the edges of the collapsed segmentation and marked this edge as the border between CSF and gray matter. We then used the unwarped (Van Der Walt et al., 2014) phase image of the 2D FLASH (without saturation) to determine the boundary between gray and white matter. Since the phase is sensitive to T2*, we anticipated a slight phase jump between white and gray matter (Fig. 5B). Subsequently, we counted the number of voxels between the CSF and phase jump and marked them as pure gray matter voxels across cortical depth. If a clear phase jump was not found at the white matter border, we used the Freesurfer reconstruction from the MP2RAGE anatomical scan to estimate the gray matter thickness.

#### 2.6.6 Multi-echo ICA denoising

In order to improve signal-to-noise and to remove nuisance signals, we applied multi-echo independent component analysis (ME-ICA) denoising. We used the TEDANA v24.0.2 (DuPre et al., 2021) toolbox voxels to remove non-BOLD signals from the data. We provided a binary mask that contains all brain, and used*tedana_workflow*with default options except*-fastica 15*and*masktype “decay.”*

#### 2.6.7 Temporal filtering and converting signal to percent change

We used a small temporal filter to reduce thermal noise by employing a running average filter using the*LN_TEMPSMOOTH*from the LAYNII package (using*-box*option with a kernel size of 7 TRs) (Huber, Poser, et al., 2021). We then normalized the time courses to percent signal change.

#### 2.6.8 Definition of laminar profiles

We assigned the voxels at different relative cortical depth to cortical layers based on the histology of human M1 (Palomero-Gallagher & Zilles, 2019). This allowed us to create individual time courses for the cortical surface, LII/III, LV, and LVI for each subject (Fig. 4Aand4D). We used linear interpolation for the gray matter defined insection 2.6.5to resample the time courses of each subject into 24 voxels before assigning them to a cortical layer.

#### 2.6.9 Characterizing time courses and determining onset times

To characterize the shape of the time courses, we conducted a simple numerical search using MATLAB’s*find*function to identify the time point at which the percent signal change reached its maximum amplitude at each cortical depth for each subject. We applied a strong temporal filter (gamma = 3 s) on the raw time courses to reduce the effect of high-frequency noise, and used MATLAB’s*find*function to identify the first index where the signal was above 95%, 50%, and 10% of the signal strength at the time point of the maximum amplitude.

We also fitted a polynomial curve to the raw line-scan data to find laminar onset times of the hemodynamic response (HRF) using MATLAB’s*curvefit*toolbox. We followed the procedure used inAlbers et al. (2018)using an HRF model fromBoynton et al. (1996):

$$h\left(t\right)=A*\frac{{\left(t-T_{0}\right)}^{a-1}*b^{a}}{gamma\left(a\right)}*exp\left(-{\left(t-T_{0}\right)}^{b}\right)\text{ for t}\geq T_{0}$$

$$h\left(t\right)=0\text{for t}<T_{0}.$$

We used the following constraints for the fit: 0 <A< 40; 0.1 < T_0_< 3.5; 2 <a< 4; 0.5 <b< 3. We used unfiltered, trial-averaged (8 trials), and subject-averaged (n = 10) raw time courses, selecting the interval from the onset to the offset of the tapping stimulus (20 s) to get the best estimation for the onset time T_0_at each cortical depth.

## 3 Results

### 3.1 Signal saturation

**Fig. 6.**
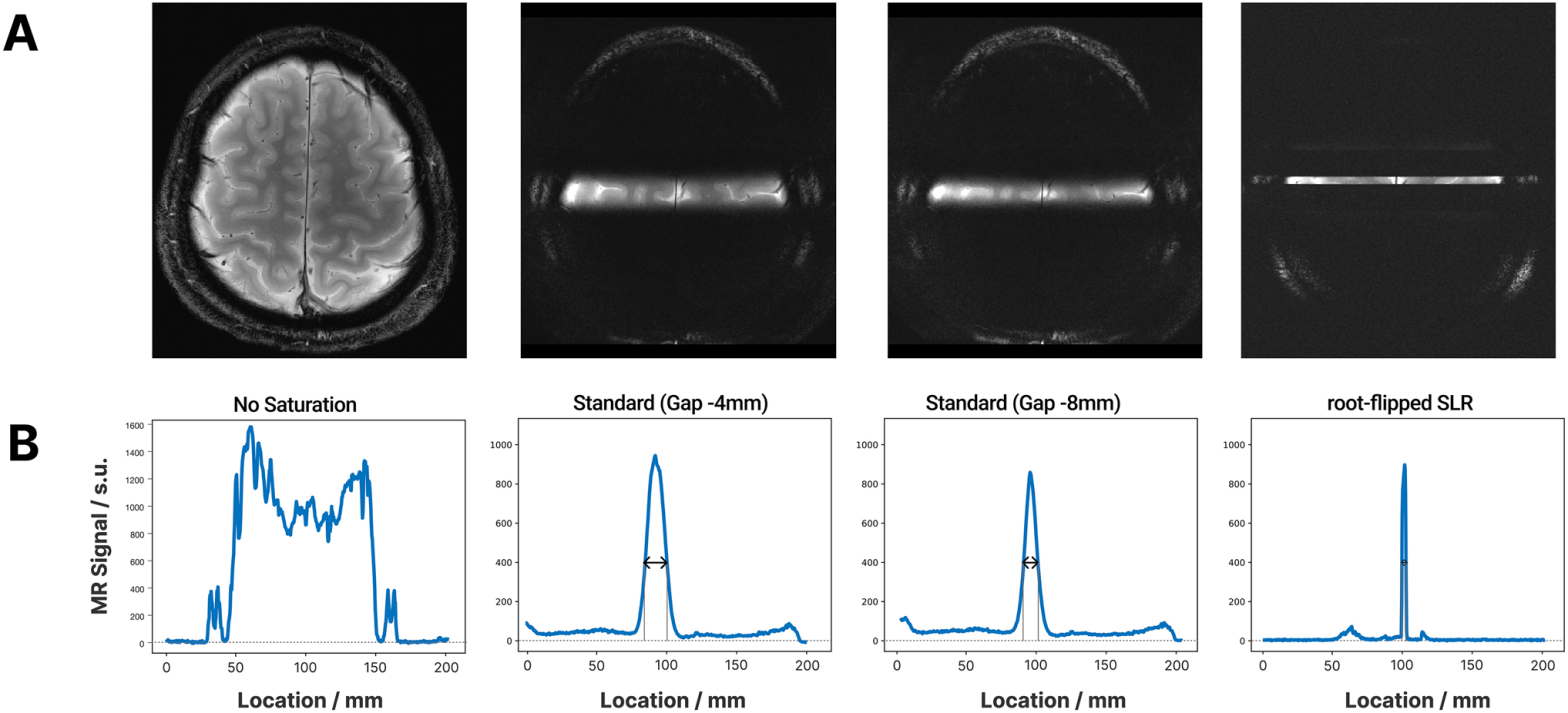
FLASH sequence using different signal saturation strategies. Panel (A): MRI image of the same slice using the following methods: no saturation, standard saturation regions (nominal gap -4 mm), standard saturation regions (nominal gap -8 mm), SLR pulses. Panel (B): Line profile across the center for each image indicating the width of the line. Spatial resolution: 0.39 x 0.39 x 3.0 mm^3^, TE/TR: 25 ms/250 ms

To evaluate the performance of the SLR pulses for selecting the line, we compared it with conventional saturation strategies.Figure 6shows the signal saturation and line sharpness for several implementations of the line-scanning sequence. The SAR levels were (from left to right) 2%, 14%, 14%, 46%. The FWHM were (from left to right) 10.9 mm, 7.4 mm, 3.9 mm. To achieve the linewidths of 10.9 and 7.4 mm using the conventional saturation approach, we needed to nominally overlap (-4 and -8 mm, respectively) the saturation slabs with respect to the targeted line width (3 mm). This makes it problematic to define signal leakage with respect to the target for the conventional approach. Therefore, we calculated the leakage as the area under the curve using the voxels outside the FWHM, divided by the total area using all voxels. The leakage values for this comparison were (from left to right) 24.2%, 18.2%, 2.1%. If we reference to the target linewidth, leakage is considerably higher, and close to 100% for the conventional saturation pulses. For the conventional approach, further reduction of the linewidth would lead to substantial reductions in signal amplitude within the line.

### 3.2 Representative example of line-scanning data in M1 during finger tapping

**Fig. 7.**
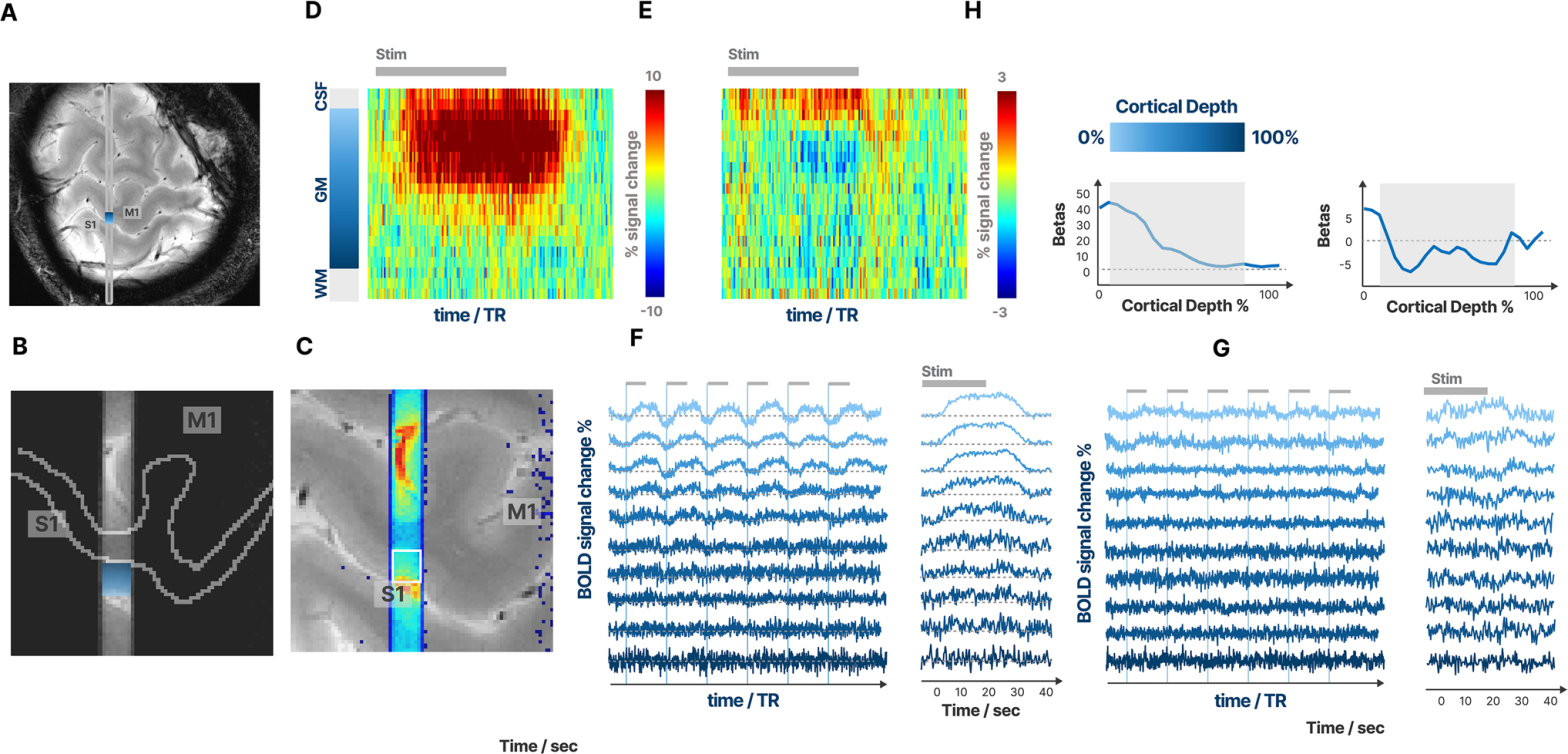
Laminar time courses in a representative participant. Panel (A) displays the magnitude image of the 2D FLASH scan with the saturation pulses turned off. The slice contains the line passing through the center, with a small ROI marking the targeted cortical patch in M1. Panel (B) shows a zoomed-in version of this line where the saturation pulses were turned on. All signals outside the line drop to zero, and the line passes perpendicularly through the WM surface which is indicated as an outline. In Panel (C), the image with saturation turned on (in color) is overlaid on the image without saturation, with the targeted ROI marked in a small box. Panels (D and E) display the trial-averaged functional activation across the cortical layers in the marked region during contralateral (D) and ipsilateral (E) tapping. The y-axis indicates cortical depth (0% at cortical surface and 100% at the white matter border), and the x-axis indicates time from 0 to 40 s. The BOLD activity peaks at the pial surface and reduces in amplitude with increasing cortical depth. Panels (F and G) show the percent signal change of the laminar time courses in the targeted patch during six trials, sorted by cortical depth, together with the stimulus onsets of the experiment (Panel F: contralateral. Panel G: ipsilateral). Next to the plot, we also show the trial-averaged time courses of the data from the same panel. The gray bar above the plot shows the 20 s stimulus cue for the finger tapping. The upper layers show the strongest BOLD signal, with the signal of the deep layers dropping to 0% signal change. Panel (H) shows the betas for all voxels across cortical depth, showing that the betas also drop to zero with increasing cortical depth.

Figure 7shows a typical line-scan experiment conducted during finger tapping in a representative participant. The line passes through the M1/S1 region associated with the hand representation. After identifying the gray matter area (seesection 2.6.5), we located nine gray matter voxels.Figure 7D-Gshows the signal from gray matter and a few extra voxels at the CSF and white matter border. As expected, the strongest amplitude of the BOLD responses was found in the upper layers at the CSF border, as indicated by the time course and beta values for this region.

### 3.3 Cortical depth analysis

**Fig. 8.**
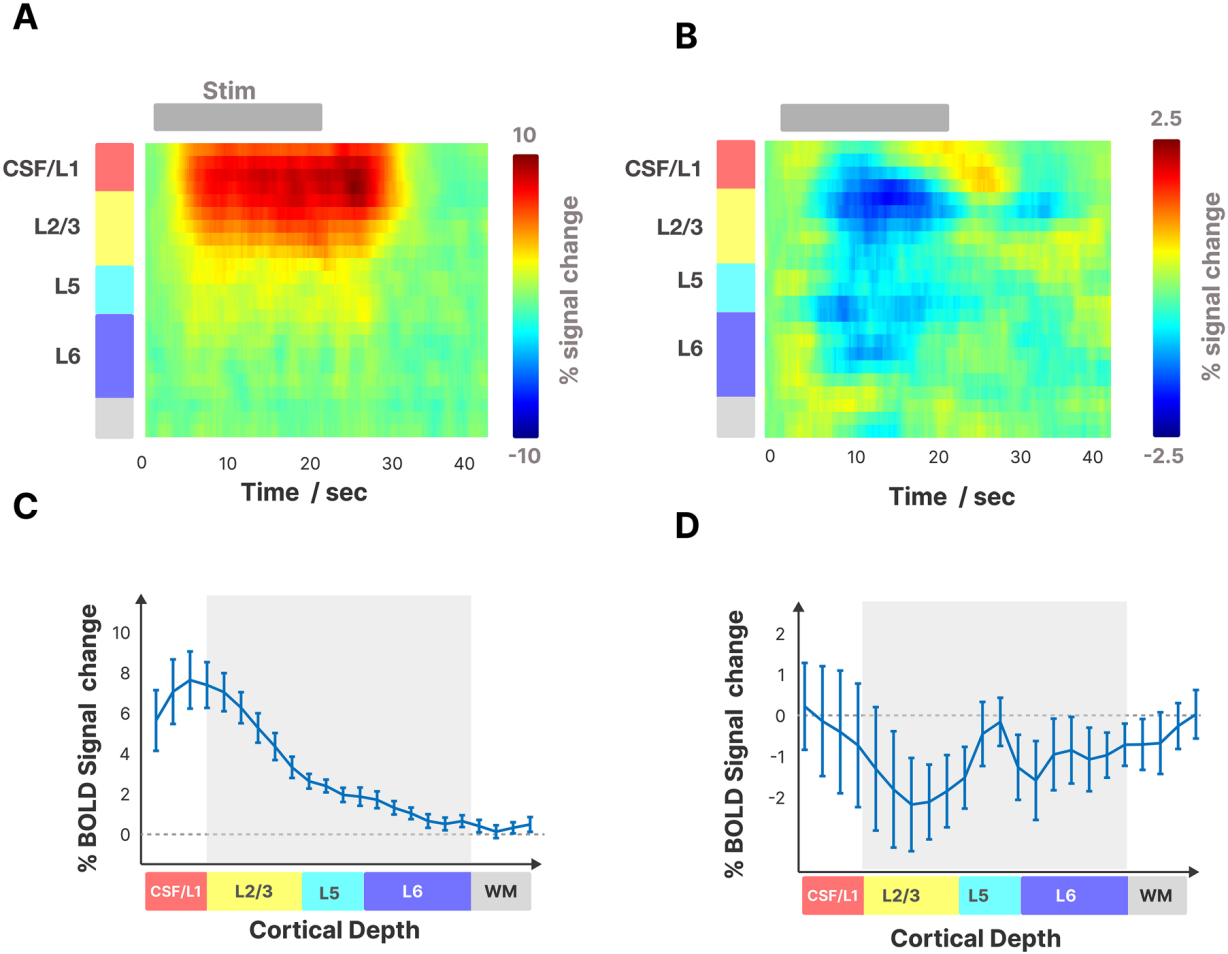
Panels show the time courses of the BOLD response to contralateral (A) and ipsilateral (B) finger tapping, averaged across subjects and trials for the layers in M1. The x-axis ranges from 0 to 40 s and the y-axis shows the cortical depth from top to bottom. CSF is on top, and the white matter is on the bottom. Panels (C and D) show the laminar profile with the mean percent signal change across the cortical ribbon (n = 10, mean + sem), 10 s after stimulus onset. The negative BOLD appears inside the gray matter, while the positive BOLD appears mainly at the cortical surface.

**Fig. 9.**
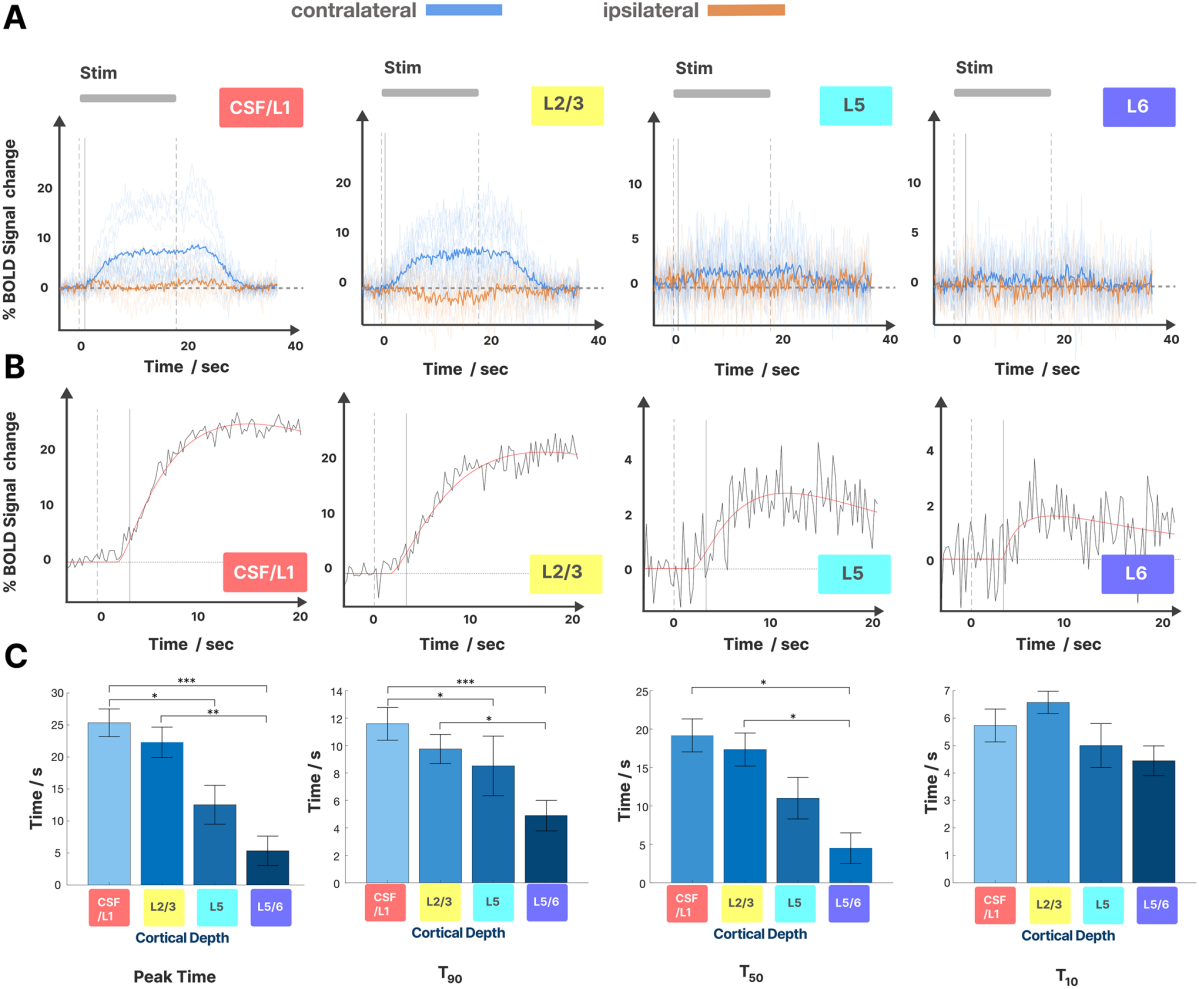
Laminar time courses and fitting of onset times at different cortical depths in the primary motor cortex (0–20% depth: CSF, 20–49%: L2/3, 50–67% L5, 67–100%: L6). (A) Trial average response during contralateral (blue) and ipsilateral (orange) finger tapping. The thick line is the group average, and the thin lines are individual data. The stimulus interval is marked with a gray bar (20 s duration, onset and offset marked with dotted lines). (B) Result of the fitting procedure with mean raw data (black) and polynomial fit (red). (C) Timings derived from the average time courses across subjects during contralateral tapping. We could find significant differences (p < 0.05) for peak times between upper and deeper layers (L1/CSF-L6, p = 0.0003; L1/CSF-L5, p = 0.009; L2/3-L6, p = 0.002; L2/3-L5, p = 0.02) when the amplitude reached 50% and 90% (T_50_, T_90_) of the maximum amplitude. All p-values were Bonferri corrected for multiple comparisons.

The mean cortical thickness of our patch in M1 was 3.3 ± 0.2 mm and spanned on average 8.5 ± 0.4 voxels (n = 10).Figure 8shows the mean time courses from our line-scanning experiment across the cortical depth, comparing tapping with the contralateral and ipsilateral hand. We found that contralateral tapping evoked the strongest BOLD response just above the pial surface (Fig. 8A). We observed BOLD responses with smaller amplitude in the gray matter. The time courses in the gray matter region near the cortical surface (around L2/3) did not reach plateau but rose slowly reaching 95% of their maximum amplitude toward the end of the stimulus period at 22.0 ± 1.9 s after onset (n = 10) (Fig. 9). In contrast, deeper layers plateaued and reached 95% of the maximum amplitude at 9.8 ± 2.4 s after stimulus onset. During ipsilateral tapping, we observed a negative BOLD response that peaked in the middle of the cortex (Fig. 8B) while the surface was inactive or had a slight positive BOLD. We also noted that the deeper layers reached 95% of the maximum negative BOLD earlier (11.2 + 1.5 s) than the gray matter closer to the cortical surface (15.3 ± 1.5 s). The negative BOLD returned to baseline faster after stimulus offset than the positive BOLD in all cortical layers.

### 3.4 Time course analysis

Figure 9Ashows the trial-averaged time courses of each cortical depth for both ipsilateral and contralateral tapping.Figure 9Bshows the mean response and the results from the fitting process are described insection 2.6.9.Figure 9Csummarizes the results of the analysis of temporal properties for T10, T50, and T90 when the signal reached 10%, 50%, and 90% of the peak value in each cortical depth.

The results of the fitting process for the onset times are displayed inTable 2.

**Table 2.** Estimated onset times during contralateral tapping (n = 10) as a result of a double-exponential fit (Albers et al., 2018).

	0-20% (Surface)	20-49% (L2/3)	49-67% (L5)	67-100% (L6)
T _0_	1.1 s R ^2^ = 0.97	0.7 s R ^2^ = 0.93	1.0 s R ^2^ = 0.49	2.3 s R ^2^ = 0.22

## 4 Discussion

We present a line-scanning fMRI method for obtaining highly localized laminar BOLD responses in a selected location of the human cortex. Our implementation has low SAR demands and achieves a sharp line profile, which was used to perform layer-specific fMRI in humans with high spatiotemporal resolution. By placing the line on the primary motor cortex (M1) during a finger-tapping task, we demonstrate its ability to sample distinct BOLD time courses across cortical depth. This makes the method a promising complementary tool for neuroscientific and clinical applications of high-resolution fMRI.

Several studies have found differences in the time courses between layers and paradigms, although mostly in primary sensory cortex.Jin and Kim (2008)reported laminar differences in onset times and time-to-peak of BOLD and CBV-weighted recordings.Tian et al. (2010)showed that the hemodynamic response has the earliest onset in layer IV, suggesting it begins in layer IV and propagates to the superficial layers.Yacoub et. al. (2006)also showed different time courses in superficial vessels and gray matter in the cat, whileBohraus et al. (2023)found similar laminar differences in the time courses between BOLD, CBF, and CMRO2 in macaques. Insights from animal studies using electrophysiological and oxygen measurements (Bentley et al., 2016) suggest that the shape of BOLD time courses may be modulated by various underlying mechanisms, which may also differ between cortical areas and layers.Shmuel et al. (2006)andHe et al. (2022)studied HRFs of negative BOLD signals and found that they are significantly different from positive BOLD signals, suggesting that they could be caused by different underlying neural or vascular dynamics.de la Rosa et al. (2021)also noted that the HRF of negative BOLD significantly differs from the HRF of positive BOLD, and is not just its inverted version.Goense et al. (2012)reported that negative BOLD in macaque V1 has different laminar profiles, suggesting that different neurovascular coupling mechanisms operate at different cortical depths.Kashyap et al. (2018)used localized fMRI at a very high spatial resolution in humans and found the BOLD response within gray matter had a stronger post-stimulus undershoot than closer to the pial surface. In summary, differences in the time courses between layers and between paradigms are common. Although most of the studies focussed on primary sensory or visual cortex, we recorded time courses in M1 and also found different laminar time courses between positive and negative BOLD. Our method provides a tool to study these differences in humans with high laminar and tangential precision.

Line-scanning fMRI of cortical layers was first applied in animals (Yu et al., 2014). They made use of its high spatiotemporal resolution and measured laminar specificity of the BOLD response in the rat cortex (Choi et al., 2023,^2024^). In human studies, line-scanning fMRI is a relatively new frontier (Morgan et al., 2020;Raimondo et al., 2021). The SAR limitations in humans are more stringent than those in animals, which makes it challenging to achieve GE-based line scanning with saturation RF pulses that have both a high bandwidth-time product and a very short TR.Raimondo et al. (2021)introduced a line-scanning GE sequence using a standard saturation scheme, with a line with FWHM of ~6 mm, which works in V1 or where activation is extensive, but may cause blurring by including BOLD and nuisance signals from non-target areas when the tangential spread of the activation is small. When we reduced the linewidth to ~4 mm using the conventional saturation pulses, there was a high signal loss in our target line, indicating the challenge of GE-based line scanning of small target areas or where sharp lines are needed. Spin-echo line scanning dramatically improves the sharpness of the line (Raimondo, Heij, et al., 2023), but at the cost of SNR and temporal resolution. They demonstrated that SE line scanning can create sharp line profiles, making this a promising approach with a clear line selection.

The approach we present here involves slightly longer TR due to the extra saturation pulses, but achieves a very sharp line profile (FWHM of 3.9 mm, seesection 3.1) that maintains signal within the line (97% of signal coming from the line, seesection 3.1) and reduces the contribution of signals from outside the line (2.2% leakage signal from outside the line). Fat signals did not tend to leak into our line in M1, but that could be different for other brain regions. Although there is no risk that fat produces functional signals, it could introduce signal instabilities. We recommend to apply fat saturation in those cases even though it may increase TR. The smallest possible TR with our GE-based sequence was 109 ms. This TR leads, however, to high SAR and reduced SNR. We, therefore, chose a slightly longer TR than other studies (Raimondo et al., 2021). Sequence improvements such as ramp sampling, stronger gradients or lower gradient spoil moments, and/or RF pulse schemes with reduced SAR demand could potentially lower the TR in the future. Regarding the spatial resolution limit, the sequence is capable of voxel sizes down to around 0.1 mm, but such voxel sizes may be more susceptible to head motion.

We demonstrate the potential of GE-line scanning by using it to extract laminar fMRI signals in the hand area of M1 while participants were performing a motor task. Our method successfully detected positive and negative BOLD time courses, and revealed differences across cortical depth. We chose this stimulus as it is widely used and known for creating strong BOLD amplitudes, and because we expected to see differences in the time courses (Schäfer et al., 2012). For the positive BOLD response, we observed a peak at the cortical surface and a faint “shoulder” in the deeper layers, which is in agreement with previous high spatial resolution studies at lower temporal resolution (Huber et al., 2017;Knudsen et al., 2023;Shao et al., 2021). For the negative BOLD response, we see a trend of a negative “double peak” with the signal peaking inside the cortex twice, with a clear negative peak in the upper layers (around L1/2, 0–20% cortical depth) and a weaker peak in the deeper layers (L6, 67–100%). This could correspond to neural inhibition (Stefanovic et al., 2004) with the upper “input” layers and the deeper “output” layers of M1 receiving transcallosal inhibitory input (Persichetti et al., 2020). There is also a hint of a post-stimulus laminar pattern for the negative BOLD which could relate toMullinger et al.’s (2017)findings that suggest underlying inhibitory neural process. We think this could be explored in a future study using a stimulus paradigm tailored to elicit post-stimulus responses, perhaps in combination with a CBF-weighted reference. The pattern we observe may be in line with findings fromHuber et al. (2017), considering that their cortical activation shows large variations across M1 and not all areas in BA4a seem to have a “double-peak” pattern. However, other studies report a negative BOLD response during ipsilateral tapping (Mullinger et al., 2014;Newton et al., 2005;Stefanovic et al., 2004;Yuan et al., 2011), although at lower spatial resolution. We speculate that the difference could be explained by some variability of the experimental design, assumptions of the HRF shape using a GLM, age dependency, or by individual differences. Further studies, especially a CBV-weighted or CBF-weighted line-scanning implementation such as VASO (Huber et al., 2017) or VAPER (Chai et al., 2020) may confirm this. We also note a high percent signal change with a few participants toward the cortical surface. We explain this by proximity to superficial pial veins or larger draining veins in these participants, (seeBause et al., 2020). We did not specifically select areas without large draining veins for our line positioning, and included all voxels in our analysis. In future studies, we aim to choose alternative locations during line positioning further away from large veins to reduce this bias.

The main limitation for GE line scanning is head motion (Raimondo, Priovoulos, et al., 2023), and our method is affected by this problem too. When participants move their heads even slightly (>0.3 mm) after the alignment process, the assignment of a time course to a cortical depth at a specific location is lost and cannot be recovered from the data. We, therefore, relied on recruiting experienced subjects, strong head fixation with cushions, training of participants to keep their head in the same position, and running the experiment as quickly as possible. This left us a window of maximum 20 min to collect the functional data while more standardized high-resolution scannings involve typically >1 h of scanning. We note that this could be seen also as an advantage as subjects can keep their attention high for the task. In addition, the demand for storing and processing the data is negligible in comparison with submillimeter 3D scanning with millions of voxels. We had to exclude data due to motion, especially in the last 10 min where we collected data for the negative BOLD.Raimondo, Priovoulos, et al. (2023)suggested prospective motion correction to tackle this problem, but the authors reported unwanted T1-transient signals due to their navigators. Another limitation of our method is that it reaches the limits of SAR for human scanners at very short TRs. Our current setup reaches the SAR limit around TR = 200 ms depending on the participant’s body height/weight. Future optimizations would need to find ways to reduce TR, which would be beneficial for experiments that study laminar onset times as they would be in the expected range of 100–150 ms across the layers based on animal research (Yu et al., 2014). We also noted that not all of our participants were right handed, presenting a potential cofound for the interpretation of our data.

Another improvement would be a more optimizing saturation strategies as suggested by Cloos et al. (2024) to achieve shorter TRs and improved SNR, or to explore different contrasts (spin echo, MT weighted, SSFP fMRI, diffusion fMRI, VASO/VAPER). Other optimizations will focus on ways to reduce motion problems by head fixation strategies, external motion cameras, or more optimal sequence-based navigators to decide which data or trials to reject due to motion. In addition, line-scanning data could benefit from model-based physiological noise removal techniques such asAgrawal (2020)which may reduce pulsation-related artifacts and further improve signal denoising.

## 5 Conclusion

We demonstrate the implementation of a GE-based line-scanning method for human fMRI with a narrow FWHM (3.9 mm) at high spatiotemporal resolution (voxel size 0.39 x 3.0 x 3.0 mm, TR = 250 ms). Our findings demonstrate the suitability of the method for laminar fMRI by extracting time courses across the cortical depth of the hand knob in the primary motor cortex during a finger-tapping task. The results reveal distinct temporal properties across cortical depths and paradigms. The ability to detect subtle differences in the hemodynamic response across cortical depths opens up new possibilities for non-invasively studying neural dynamics in humans.

## Data Availability

We will make code and data available upon reasonable request.

## References

[b71] Agrawal , U. , Brown , E. N. , & Lewis , L. D. ( 2020 ). Model-based physiological noise removal in fast fMRI . NeuroImage , 205 , 116231 . 10.1016/j.neuroimage.2006.01.015 31589991 PMC6911832

[b1] Albers , F. , Schmid , F. , Wachsmuth , L. , & Faber , C . ( 2018 ). Line scanning fMRI reveals earlier onset of optogenetically evoked BOLD response in rat somatosensory cortex as compared to sensory stimulation . NeuroImage , 164 , 144 – 154 . 10.1016/j.neuroimage.2016.12.059 28012967

[b2] Balasubramanian , M. , Mulkern , R. V. , Neil , J. J. , Maier , S. E. , & Polimeni , J. R . ( 2021 ). Probing in vivo cortical myeloarchitecture in humans via line-scan diffusion acquisitions at 7 T with 250-500 micron radial resolution . Magnetic Resonance in Medicine , 85 ( 1 ), 390 – 403 . 10.1002/mrm.28419 32738088 PMC7951328

[b3] Bause , J. , Polimeni , J. R. , Stelzer , J. , In , M.-H. , Ehses , P. , Kraemer-Fernandez , P. , Aghaeifar , A. , Lacosse , E. , Pohmann , R. , & Scheffler , K . ( 2020 ). Impact of prospective motion correction, distortion correction methods and large vein bias on the spatial accuracy of cortical laminar fMRI at 9.4 Tesla . NeuroImage , 208 , 116434 . 10.1016/j.neuroimage.2019.116434 31812715

[b4] Bentley , W. J. , Li , J. M. , Snyder , A. Z. , Raichle , M. E. , & Snyder , L. H . ( 2016 ). Oxygen level and LFP in task-positive and task-negative areas: Bridging bold fMRI and electrophysiology . Cerebral Cortex , 26 ( 1 ), 346 – 357 . 10.1093/cercor/bhu260 25385710 PMC4677981

[b5] Bergmann , J. , Petro , L. S. , Abbatecola , C. , Li , M. S. , Morgan , A. T. , & Muckli , L . ( 2024 ). Cortical depth profiles in primary visual cortex for illusory and imaginary experiences . Nature Communications , 15 ( 1 ), 1002 . 10.1038/s41467-024-45065-w PMC10837448 38307834

[b6] Blazejewska , A. I. , Fischl , B. , Wald , L. L. , & Polimeni , J. R . ( 2019 ). Intracortical smoothing of small-voxel fMRI data can provide increased detection power without spatial resolution losses compared to conventional large-voxel fMRI data . NeuroImage , 189 , 601 – 614 . 10.1016/j.neuroimage.2019.01.054 30690157 PMC6668026

[b72] Bohraus , Y. , Merkle , H. , Logothetis , N. K. , & Goense , J. ( 2023 ). Laminar differences in functional oxygen metabolism in monkey visual cortex measured with calibrated fMRI . Cell Reports , 42 ( 11 ), 113341. 10.1016/j.celrep.2023.113341 37897728

[b7] Boynton , G. M. , Engel , S. A. , Glover , G. H. , & Heeger , D. J . ( 1996 ). Linear systems analysis of functional magnetic resonance imaging in human v1 . The Journal of Neuroscience , 16 ( 13 ), 4207 – 4221 . 10.1523/JNEUROSCI.16-13-04207.1996 8753882 PMC6579007

[b8] Brett , M. , Markiewicz , C. J. , Hanke , M. , Côté , M.-A. , Cipollini , B. , McCarthy , P. , Jarecka , D. , Cheng , C. P. , Larson , E. , Halchenko , Y. O. , Cottaar , M. , Ghosh , S. , Wassermann , D. , Gerhard , S. , Lee , G. R. , Baratz , Z. , Wang , H.-T. , Papadopoulos Orfanos , D., Kastman , E. , … freec84 . ( 2023 ). Nipy/nibabel: 5.2.0 [Computer software]. Zenodo. 10.5281/ZENODO.10363247

[b9] Chai , Y. , Li , L. , Huber , L. , Poser , B. A. , & Bandettini , P. A . ( 2020 ). Integrated VASO and perfusion contrast: A new tool for laminar functional MRI . NeuroImage , 207 , 116358 . 10.1016/j.neuroimage.2019.116358 31740341 PMC11801192

[b10] Choi , S. , Chen , Y. , Zeng , H. , Biswal , B. , & Yu , X . ( 2023 ). Identifying the distinct spectral dynamics of laminar-specific interhemispheric connectivity with bilateral line-scanning fMRI . Journal of Cerebral Blood Flow & Metabolism , 43 ( 7 ), 1115 – 1129 . 10.1177/0271678X231158434 36803280 PMC10291453

[b11] Choi , S. , Hike , D. , Pohmann , R. , Avdievich , N. , Gomez-Cid , L. , Man , W. , Scheffler , K. , & Yu , X . ( 2024 ). Alpha-180 spin-echo-based line-scanning method for high-resolution laminar-specific fMRI in animals . Imaging Neuroscience , 2 , 1 – 14 . 10.1162/imag_a_00120 PMC12247619 40800297

[b12] Choi , S. , Yu , X. , Scheffler , K. , & Herz , K. ( n.d. ). Simultaneous acquisition of GRE- and SE-type resting-state fMRI signals with GRASE-based line-scanning in the human brain . ISMRT 31st Annual Meeting , Abstract 1105. 10.58530/2022/1105

[b13] Choi , S. , Zeng , H. , Chen , Y. , Sobczak , F. , Qian , C. , & Yu , X . ( 2022 ). Laminar-specific functional connectivity mapping with multi-slice line-scanning fMRI . Cerebral Cortex , 32 ( 20 ), 4492 – 4501 . 10.1093/cercor/bhab497 35107125 PMC9574235

[b14] Cloos , M. , Hodono , S. , Balasubramanian , M. , & Polimeni , J. ( n.d. ). Rapid gradient-recalled echo line scanning with crisp line profiles for human MRI at 7 Tesla . ISMRM & ISMRT Annual Meeting & Exhibition , Abstract 3294. 10.58530/2024/3294

[b16] De La Rosa , N. , Ress , D. , Taylor , A. J. , & Kim , J. H . ( 2021 ). Retinotopic variations of the negative blood-oxygen-level dependent hemodynamic response function in human primary visual cortex . Journal of Neurophysiology , 125 ( 4 ), 1045 – 1057 . 10.1152/jn.00676.2020 33625949 PMC8282228

[b17] Ding , S. , Royall , J. J. , Sunkin , S. M. , Ng , L. , Facer , B. A. C. , Lesnar , P. , Guillozet-Bongaarts , A. , McMurray , B. , Szafer , A. , Dolbeare , T. A. , Stevens , A. , Tirrell , L. , Benner , T. , Caldejon , S. , Dalley , R. A. , Dee , N. , Lau , C. , Nyhus , J. , Reding , M. , … Lein , E. S. ( 2016 ). Comprehensive cellular-resolution atlas of the adult human brain . Journal of Comparative Neurology , 524 ( 16 ), 3127 – 3481 . 10.1002/cne.24080 27418273 PMC5054943

[b18] DuPre , E. , Salo , T. , Ahmed , Z. , Bandettini , P. , Bottenhorn , K. , Caballero-Gaudes , C. , Dowdle , L. , Gonzalez-Castillo , J. , Heunis , S. , Kundu , P. , Laird , A. , Markello , R. , Markiewicz , C. , Moia , S. , Staden , I. , Teves , J. , Uruñuela , E. , Vaziri-Pashkam , M. , Whitaker , K. , & Handwerker , D . ( 2021 ). TE-dependent analysis of multi-echo fMRI with tedana . Journal of Open Source Software , 6 ( 66 ), 3669 . 10.21105/joss.03669

[b73] Finn , E. S. , Huber , L. , Jangraw , D. C. , Molfese , P. J. , & Bandettini , P. A. ( 2019 ). Layer-dependent activity in human prefrontal cortex during working memory . Nature Neuroscience , 22 ( 10 ), 1687 – 1695 . 10.1038/s41593-019-0487-z 31551596 PMC6764601

[b19] Fischl , B . ( 2012 ). Freesurfer . NeuroImage , 62 ( 2 ), 774 – 781 . 10.1016/j.neuroimage.2012.01.021 22248573 PMC3685476

[b20] Fracasso , A. , Luijten , P. R. , Dumoulin , S. O. , & Petridou , N . ( 2018 ). Laminar imaging of positive and negative BOLD in human visual cortex at 7 T . NeuroImage , 164 , 100 – 111 . 10.1016/j.neuroimage.2017.02.038 28213112

[b74] Goense , J. ( 2018 ). Resolving memory circuits with layer-dependent fMRI . Neuron , 99 ( 6 ), 1107 – 1109 . 10.1016/j.neuron.2018.09.004 30236280

[b21] Goense , J. , Merkle , H. , & Logothetis , N. K . ( 2012 ). High-resolution fMRI reveals laminar differences in neurovascular coupling between positive and negative bold responses . Neuron , 76 ( 3 ), 629 – 639 . 10.1016/j.neuron.2012.09.019 23141073 PMC5234326

[b22] Guidi , M. , Huber , L. , Lampe , L. , Gauthier , C. J. , & Möller , H. E . ( 2016 ). Lamina-dependent calibrated BOLD response in human primary motor cortex . NeuroImage , 141 , 250 – 261 . 10.1016/j.neuroimage.2016.06.030 27364473

[b23] Hawrylycz , M. J. , Lein , E. S. , Guillozet-Bongaarts , A. L. , Shen , E. H. , Ng , L. , Miller , J. A. , Van De Lagemaat , L. N. , Smith , K. A. , Ebbert , A. , Riley , Z. L. , Abajian , C. , Beckmann , C. F. , Bernard , A. , Bertagnolli , D. , Boe , A. F. , Cartagena , P. M. , Chakravarty , M. M. , Chapin , M. , Chong , J. , … Jones , A. R. ( 2012 ). An anatomically comprehensive atlas of the adult human brain transcriptome . Nature , 489 ( 7416 ), 391 – 399 . 10.1038/nature11405 22996553 PMC4243026

[b24] He , H. , Ettehadi , N. , Shmuel , A. , & Razlighi , Q. R . ( 2022 ). Evidence suggesting common mechanisms underlie contralateral and ipsilateral negative BOLD responses in the human visual cortex . NeuroImage , 262 , 119440 . 10.1016/j.neuroimage.2022.119440 35842097 PMC9523581

[b25] Heij , J. , Raimondo , L. , Siero , J. C. W. , Dumoulin , S. O. , Van Der Zwaag , W. , & Knapen , T . ( 2023 ). A selection and targeting framework of cortical locations for line-scanning fMRI . Human Brain Mapping , 44 ( 16 ), 5471 – 5484 . 10.1002/hbm.26459 37608563 PMC10543358

[b26] Huber , L. , Finn , E. S. , Chai , Y. , Goebel , R. , Stirnberg , R. , Stöcker , T. , Marrett , S. , Uludag , K. , Kim , S.-G. , Han , S. , Bandettini , P. A. , & Poser , B. A . ( 2021 ). Layer-dependent functional connectivity methods . Progress in Neurobiology , 207 , 101835 . 10.1016/j.pneurobio.2020.101835 32512115 PMC11800141

[b27] Huber , L. , Goense , J. , Kennerley , A. J. , Trampel , R. , Guidi , M. , Reimer , E. , Ivanov , D. , Neef , N. , Gauthier , C. J. , Turner , R. , & Möller , H. E . ( 2015 ). Cortical lamina-dependent blood volume changes in human brain at 7 T . NeuroImage , 107 , 23 – 33 . 10.1016/j.neuroimage.2014.11.046 25479018

[b28] Huber , L. , Handwerker , D. A. , Jangraw , D. C. , Chen , G. , Hall , A. , Stüber , C. , Gonzalez-Castillo , J. , Ivanov , D. , Marrett , S. , Guidi , M. , Goense , J. , Poser , B. A. , & Bandettini , P. A . ( 2017 ). High-resolution CBV-fMRI allows mapping of laminar activity and connectivity of cortical input and output in human m1 . Neuron , 96 ( 6 ), 1253 - 1263.e7 . 10.1016/j.neuron.2017.11.005 29224727 PMC5739950

[b29] Huber , L. (Renzo) , Poser , B. A. , Bandettini , P. A. , Arora , K. , Wagstyl , K. , Cho , S. , Goense , J. , Nothnagel , N. , Morgan , A. T. , Van Den Hurk , J. , Müller , A. K. , Reynolds , R. C. , Glen , D. R. , Goebel , R. , & Gulban , O. F . ( 2021 ). LayNii: A software suite for layer-fMRI . NeuroImage , 237 , 118091 . 10.1016/j.neuroimage.2021.118091 33991698 PMC7615890

[b30] Huber , L. , Uludağ , K. , & Möller , H. E . ( 2019 ). Non-BOLD contrast for laminar fMRI in humans: CBF, CBV, and CMRO2 . NeuroImage , 197 , 742 – 760 . 10.1016/j.neuroimage.2017.07.041 28736310 PMC12906290

[b31] Jia , K. , Goebel , R. , & Kourtzi , Z . ( 2023 ). Ultra-high field imaging of human visual cognition . Annual Review of Vision Science , 9 ( 1 ), 479 – 500 . 10.1146/annurev-vision-111022-123830 37137282

[b32] Jin , T. , & Kim , S.-G . ( 2008 ). Cortical layer-dependent dynamic blood oxygenation, cerebral blood flow and cerebral blood volume responses during visual stimulation . NeuroImage , 43 ( 1 ), 1 – 9 . 10.1016/j.neuroimage.2008.06.029 18655837 PMC2579763

[b75] Kashyap , S. , Ivanov , D. , Havlicek , M. , Huber , L. , Poser , B. A. , & Uludağ , K. ( 2021 ). Sub-millimetre resolution laminar fMRI using Arterial Spin Labelling in humans at 7 T . PLOS ONE , 16 ( 4 ), e0250504. 10.1371/journal.pone.0250504 PMC8075193 33901230

[b33] Kashyap , S. , Ivanov , D. , Havlicek , M. , Sengupta , S. , Poser , B. A. , & Uludağ , K . ( 2018 ). Resolving laminar activation in human V1 using ultra-high spatial resolution fMRI at 7T . Scientific Reports , 8 ( 1 ), 17063 . 10.1038/s41598-018-35333-3 30459391 PMC6244001

[b34] Kiebel , S. J. , Goebel , R. , & Friston , K. J . ( 2000 ). Anatomically informed basis functions . NeuroImage , 11 ( 6 ), 656 – 667 . 10.1006/nimg.1999.0542 10860794

[b35] Knudsen , L. , Bailey , C. J. , Blicher , J. U. , Yang , Y. , Zhang , P. , & Lund , T. E . ( 2023 ). Improved sensitivity and microvascular weighting of 3T laminar fMRI with GE-BOLD using NORDIC and phase regression . NeuroImage , 271 , 120011 . 10.1016/j.neuroimage.2023.120011 36914107

[b36] Koiso , K. , Müller , A. K. , Akamatsu , K. , Dresbach , S. , Wiggins , C. J. , Gulban , O. F. , Goebel , R. , Miyawaki , Y. , Poser , B. A. , & Huber , L . ( 2023 ). Acquisition and processing methods of whole-brain layer-fMRI VASO and BOLD: The Kenshu dataset . Aperture Neuro , 3 , 1 – 22 . 10.52294/001c.87961 PMC11981596 40206493

[b37] Kundu , P. , Inati , S. J. , Evans , J. W. , Luh , W.-M. , & Bandettini , P. A . ( 2012 ). Differentiating BOLD and non-BOLD signals in fMRI time series using multi-echo EPI . NeuroImage , 60 ( 3 ), 1759 – 1770 . 10.1016/j.neuroimage.2011.12.028 22209809 PMC3350785

[b38] Li , X. , Morgan , P. S. , Ashburner , J. , Smith , J. , & Rorden , C . ( 2016 ). The first step for neuroimaging data analysis: DICOM to NIfTI conversion . Journal of Neuroscience Methods , 264 , 47 – 56 . 10.1016/j.jneumeth.2016.03.001 26945974

[b39] Morgan , A. T. , Nothnagel , N. , Petro , Lucy. S. , Goense , J. , & Muckli , L . ( 2020 ). High-resolution line-scanning reveals distinct visual response properties across human cortical layers . bioRXiv . 10.1101/2020.06.30.179762

[b40] Mullinger , K. J. , Cherukara , M. T. , Buxton , R. B. , Francis , S. T. , & Mayhew , S. D . ( 2017 ). Post-stimulus fMRI and EEG responses: Evidence for a neuronal origin hypothesised to be inhibitory . NeuroImage , 157 , 388 – 399 . 10.1016/j.neuroimage.2017.06.020 28610902 PMC6475192

[b41] Mullinger , K. J. , Mayhew , S. D. , Bagshaw , A. P. , Bowtell , R. , & Francis , S. T . ( 2014 ). Evidence that the negative BOLD response is neuronal in origin: A simultaneous EEG–BOLD–CBF study in humans . NeuroImage , 94 , 263 – 274 . 10.1016/j.neuroimage.2014.02.029 24632092

[b42] Nassirpour , S. , Chang , P. , Fillmer , A. , & Henning , A . ( 2018 ). A comparison of optimization algorithms for localized in vivo B _0_ shimming . Magnetic Resonance in Medicine , 79 ( 2 ), 1145 – 1156 . 10.1002/mrm.26758 28543722

[b43] Newton , J. M. , Sunderland , A. , & Gowland , P. A . ( 2005 ). fMRI signal decreases in ipsilateral primary motor cortex during unilateral hand movements are related to duration and side of movement . NeuroImage , 24 ( 4 ), 1080 – 1087 . 10.1016/j.neuroimage.2004.10.003 15670685

[b44] Nothnagel , N. , Huber , L. , & Goense , J . ( 2023 ). Sequences and contrasts for non-BOLD fMRI . In Advances in Magnetic Resonance Technology and Applications (Vol. 10 , pp. 371 – 386 ). Elsevier . 10.1016/B978-0-323-99898-7.00007-9

[b45] Nunes , D. , Gil , R. , & Shemesh , N . ( 2021 ). A rapid-onset diffusion functional MRI signal reflects neuromorphological coupling dynamics . NeuroImage , 231 , 117862 . 10.1016/j.neuroimage.2021.117862 33592243

[b46] Nunes , D. , Ianus , A. , & Shemesh , N . ( 2019 ). Layer-specific connectivity revealed by diffusion-weighted functional MRI in the rat thalamocortical pathway . NeuroImage , 184 , 646 – 657 . 10.1016/j.neuroimage.2018.09.050 30267858 PMC6264401

[b47] Ong , F. , & Lustig , M. ( 2019 ). SigPy: A python package for high performance iterative reconstruction . Proceedings of the ISMRM 27th Annual Meeting, Montreal, Quebec , 4819 , 5 . https://archive.ismrm.org/2019/4819.html

[b48] Palomero-Gallagher , N. , & Zilles , K . ( 2019 ). Cortical layers: Cyto-, myelo-, receptor- and synaptic architecture in human cortical areas . NeuroImage , 197 , 716 – 741 . 10.1016/j.neuroimage.2017.08.035 28811255

[b49] Persichetti , A. S. , Avery , J. A. , Huber , L. , Merriam , E. P. , & Martin , A . ( 2020 ). Layer-specific contributions to imagined and executed hand movements in human primary motor cortex . Current Biology , 30 ( 9 ), 1721 - 1725.e3 . 10.1016/j.cub.2020.02.046 32220318 PMC7202997

[b50] Raimondo , L. , Heij , J. , Knapen , T. , Dumoulin , S. O. , Van Der Zwaag , W. , & Siero , J. C. W . ( 2023 ). Towards functional spin-echo BOLD line-scanning in humans at 7T . Magnetic Resonance Materials in Physics, Biology and Medicine , 36 ( 2 ), 317 – 327 . 10.1007/s10334-022-01059-7 PMC10140128 36625959

[b51] Raimondo , L. , Knapen , T. , Oliveira , Ĺ. A. F. , Yu , X. , Dumoulin , S. O. , Van Der Zwaag , W. , & Siero , J. C. W . ( 2021 ). A line through the brain: Implementation of human line-scanning at 7T for ultra-high spatiotemporal resolution fMRI . Journal of Cerebral Blood Flow & Metabolism , 41 ( 11 ), 2831 – 2843 . 10.1177/0271678X211037266 34415208 PMC8756483

[b52] Raimondo , L. , Priovoulos , N. , Heij , J. , Knapen , T. , Dumoulin , S. , Siero , J. , & Van Der Zwaag , W . ( n.d. ). Prospective motion correction in multi-echo line-scanning fMRI at 7T: Sequence implementation and strategies for functional data analysis . ISMRT 31st Annual Meeting , Abstract 2195. 10.58530/2022/2195

[b53] Raimondo , L. , Priovoulos , N. , Passarinho , C. , Heij , J. , Knapen , T. , Dumoulin , S. O. , Siero , J. C. W. , & Van Der Zwaag , W . ( 2023 ). Robust high spatio-temporal line-scanning fMRI in humans at 7T using multi-echo readouts, denoising and prospective motion correction . Journal of Neuroscience Methods , 384 , 109746 . 10.1016/j.jneumeth.2022.109746 36403778

[b54] Schäfer , K. , Blankenburg , F. , Kupers , R. , Grüner , J. M. , Law , I. , Lauritzen , M. , & Larsson , H. B. W . ( 2012 ). Negative BOLD signal changes in ipsilateral primary somatosensory cortex are associated with perfusion decreases and behavioral evidence for functional inhibition . NeuroImage , 59 ( 4 ), 3119 – 3127 . 10.1016/j.neuroimage.2011.11.085 22155327

[b55] Self , M. W. , Van Kerkoerle , T. , Goebel , R. , & Roelfsema , P. R . ( 2019 ). Benchmarking laminar fMRI: Neuronal spiking and synaptic activity during top-down and bottom-up processing in the different layers of cortex . NeuroImage , 197 , 806 – 817 . 10.1016/j.neuroimage.2017.06.045 28648888

[b56] Shao , X. , Guo , F. , Shou , Q. , Wang , K. , Jann , K. , Yan , L. , Toga , A. W. , Zhang , P. , & Wang , D. J. J . ( 2021 ). Laminar perfusion imaging with zoomed arterial spin labeling at 7 Tesla . NeuroImage , 245 , 118724 . 10.1016/j.neuroimage.2021.118724 34780918 PMC8727512

[b57] Shao , X. , Hua , J. , & Wang , D. J . ( 2022 ). Concurrent laminar CBF, CBV, T2-BOLD and CMRO2 fMRI at 7T in human primary motor cortex . ISMRT 31st Annual Meeting , Abstract 0404. 10.58530/2022/0404

[b58] Shen , Q. , Ren , H. , & Duong , T. Q . ( 2008 ). CBF, BOLD, CBV, and CMRO _2_ fMRI signal temporal dynamics at 500-msec resolution . Journal of Magnetic Resonance Imaging , 27 ( 3 ), 599 – 606 . 10.1002/jmri.21203 18219630 PMC2900800

[b59] Shmuel , A. , Augath , M. , Oeltermann , A. , & Logothetis , N. K . ( 2006 ). Negative functional MRI response correlates with decreases in neuronal activity in monkey visual area V1 . Nature Neuroscience , 9 ( 4 ), 569 – 577 . 10.1038/nn1675 16547508

[b60] Siero , J. C. W. , Ramsey , N. F. , Hoogduin , H. , Klomp , D. W. J. , Luijten , P. R. , & Petridou , N . ( 2013 ). Bold specificity and dynamics evaluated in humans at 7 t: Comparing gradient-echo and spin-echo hemodynamic responses . PLoS One , 8 ( 1 ), e54560 . 10.1371/journal.pone.0054560 23336008 PMC3546000

[b61] Stefanovic , B. , Warnking , J. M. , & Pike , G. B . ( 2004 ). Hemodynamic and metabolic responses to neuronal inhibition . NeuroImage , 22 ( 2 ), 771 – 778 . 10.1016/j.neuroimage.2004.01.036 15193606

[b62] Tian , P. , Teng , I. C. , May , L. D. , Kurz , R. , Lu , K. , Scadeng , M. , Hillman , E. M. C. , De Crespigny , A. J. , D’Arceuil , H. E. , Mandeville , J. B. , Marota , J. J. A. , Rosen , B. R. , Liu , T. T. , Boas , D. A. , Buxton , R. B. , Dale , A. M. , & Devor , A . ( 2010 ). Cortical depth-specific microvascular dilation underlies laminar differences in blood oxygenation level-dependent functional MRI signal . Proceedings of the National Academy of Sciences , 107 ( 34 ), 15246 – 15251 . 10.1073/pnas.1006735107 PMC2930564 20696904

[b63] Uecker , M. , Ong , F. , Tamir , J. I. , Bahri , D. , Virtue , P. , Cheng , J. Y. , Zhang , T. , & Lustig , P. ( 2015 ). Berkeley advanced reconstruction toolbox . Proceedings of the International Society for Magnetic Resonance in Medicine , 23 , 2486 . https://archive.ismrm.org/2015/2486.html

[b64] Van Der Walt , S. , Schönberger , J. L. , Nunez-Iglesias , J. , Boulogne , F. , Warner , J. D. , Yager , N. , Gouillart , E. , & Yu , T . ( 2014 ). Scikit-image: Image processing in python . PeerJ , 2 , e453 . 10.7717/peerj.453 25024921 PMC4081273

[b65] Yacoub , E. , Ugurbil , K. , & Harel , N . ( 2006 ). The spatial dependence of the poststimulus undershoot as revealed by high-resolution bold- and CBV-weighted fMRI . Journal of Cerebral Blood Flow & Metabolism , 26 ( 5 ), 634 – 644 . 10.1038/sj.jcbfm.9600239 16222242

[b66] Yu , X. , Qian , C. , Chen , D. , Dodd , S. J. , & Koretsky , A. P . ( 2014 ). Deciphering laminar-specific neural inputs with line-scanning fMRI . Nature Methods , 11 ( 1 ), 55 – 58 . 10.1038/nmeth.2730 24240320 PMC4276040

[b67] Yuan , H. , Perdoni , C. , Yang , L. , & He , B . ( 2011 ). Differential electrophysiological coupling for positive and negative bold responses during unilateral hand movements . Journal of Neuroscience , 31 ( 26 ), 9585 – 9593 . 10.1523/JNEUROSCI.5312-10.2011 21715623 PMC3142625

[b68] Yushkevich , P. A. , Piven , J. , Hazlett , H. C. , Smith , R. G. , Ho , S. , Gee , J. C. , & Gerig , G . ( 2006 ). User-guided 3D active contour segmentation of anatomical structures: Significantly improved efficiency and reliability . NeuroImage , 31 ( 3 ), 1116 – 1128 . 10.1016/j.neuroimage.2006.01.015 16545965

